# Influence of Gut Microbiota on Subclinical Inflammation and Insulin Resistance

**DOI:** 10.1155/2013/986734

**Published:** 2013-06-12

**Authors:** Bruno Melo Carvalho, Mario Jose Abdalla Saad

**Affiliations:** Internal Medicine Department, Faculty of Medical Sciences, FCM, UNICAMP, Rua Tessália Vieira de Camargo, 126 Cidade Universitária Zeferino Vaz, 13083-887 Campinas, SP, Brazil

## Abstract

Obesity is the main condition that is correlated with the appearance of insulin resistance, which is the major link among its comorbidities, such as type 2 diabetes, nonalcoholic fatty liver disease, cardiovascular and neurodegenerative diseases, and several types of cancer. Obesity affects a large number of individuals worldwide; it degrades human health and quality of life. Here, we review the role of the gut microbiota in the pathophysiology of obesity and type 2 diabetes, which is promoted by a bacterial diversity shift mediated by overnutrition. Whole bacteria, their products, and metabolites undergo increased translocation through the gut epithelium to the circulation due to degraded tight junctions and the consequent increase in intestinal permeability that culminates in inflammation and insulin resistance. Several strategies focusing on modulation of the gut microbiota (antibiotics, probiotics, and prebiotics) are being experimentally employed in metabolic derangement in order to reduce intestinal permeability, increase the production of short chain fatty acids and anorectic gut hormones, and promote insulin sensitivity to counteract the inflammatory status and insulin resistance found in obese individuals.

## 1. Introduction

Insulin resistance is the main outcome caused by nutrient overload, lipids, infections, and sepsis-induced inflammation that affects insulin-sensitive tissues, such as the liver, muscle, adipose tissue, and hypothalamus, and which also promotes defects in cell signaling pathways and homeostasis [1]. The ingestion of an unbalanced diet and low physical activity observed in recent years in the global population are the main drivers of the epidemic rates of obesity reached in the past few decades [2]. A prospective study evaluated more than 9 million people worldwide over the last three decades and observed that, globally, the average body mass index (BMI) increased by 0.4-0.5 kg/m^2^ per decade; moreover, subregion trends showed that the average BMI increased by 1.4 kg/m^2^ in men and 1.9 kg/m^2^ in women, per decade [3]. However, not only developed countries such as the United States are affected by this epidemic of obesity, but also countries under development, such as Brazil and other countries, are also affected in a similar way [4].

The World Health Organization has observed that more than 1.4 billion adults are overweight and, of these, at least 200 million men and 300 million women are clinically obese [5]. Some studies have shown that the increased rate of obesity has slowed down over the last five years, trended by some specific population groups, in eastern Europe, South America, and even in some specific population groups in the United States. However, the prevalence of obesity remains high and the health costs associated with obese individuals are huge, ranging from 2 to 7% of the health budgets in high income countries, which is followed by the low income nations, while the mortality among obese people is increased [3, 6, 7].

Obesity is characterized by chronic subclinical inflammation that affects insulin activity in its metabolically sensitive tissues, notably the liver, muscle, and adipose tissue, and drives a metabolic disorder that culminates in the deregulation of glucose homeostasis [8]. Since the observation that obese adipose tissue shows increased expression of the proinflammatory cytokine TNF-*α* [9], extensive research efforts have been made regarding inflammation in metabolic tissues, trying to determine what primes the insulin resistance phenomenon, the consequences of obesity, how to improve the molecular knowledge of insulin activity attenuation mediated by obesity, and how it can be managed in order to improve patient quality of life. Attempts have also been made to reduce or prevent the incidence of obesity and its comorbidities.

Here, we will focus on reviewing the latest contributions to the literature on the influence of the intestine on the pathogenesis of insulin resistance and the consequences of insulin resistance on the liver, muscle, adipose tissue, and hypothalamus, as well as the mechanisms by which the gut microbiota influences systemic insulin resistance.

## 2. Intestinal Participation in Insulin Resistance

For several decades, it has been known that the mammalians intestine harbors a great number of bacteria (~10^14^ bacteria) that even surpasses the total number of cells that comprise mammalian tissues, systems, and, ultimately, the entire body [10]. Furthermore, it is estimated that the gut microbiota contains, at least, 100-fold more genes than the mammalian genome. These bacteria can live in a symbiotic way but, in certain cases, promote disease [11]. However, over the last ten years, this community that lives within the mammalian body has been gaining the status of a microbial organ that contributes to homeostasis and impacts directly on energy metabolism and insulin sensitivity [12, 13].

## 3. The Obese Microbiome Has Increased Energy Harvesting Ability

One of the first and foremost important observations on the role of the gut microbiota in insulin sensitivity and body weight management was observed when *germ-free* mice that were infected with the gut microbiota content of a conventionally raised mice showed an increase (by about 60%) in body fat content. Moreover, the occurrence of insulin resistance and glucose intolerance was seen within 14 days, even with a reduction in food intake (standard chow), providing novel evidence that the bacteria community, in some way, controlled energy metabolism [14]. Additionally, it was described that the activity of a lipoprotein lipase (LPL) suppressor, known as fasting-induced adipocyte factor (Fiaf) or angiopoietin-like protein 4 (ANGPTL4), controlled the fat storage abnormalities observed in conventionalized *germ-free* mice, where the gut microbiota induced selective suppression of this protein in intestinal cells and promoted an increase in LPL activity. This resulted in increased triglyceride storage in adipocytes, which was prevented upon the conventionalization of Fiaf^−/−^ *germ-free* mice [14].

It was also shown that the resistance of *germ-free* mice against diet-induced obesity relied on increased liver and skeletal muscle AMPK activity and its downstream targets. This induced the activation of fatty acid oxidation and increased energy expenditure, which regulated body weight gain, since Fiaf expression in the gut epithelium was highly suppressed in *germ-free* mice fed a high-fat diet, keeping the body weight almost unaltered, a feature that was not maintained in Fiaf^−/−^ *germ-free* mice [15].

The great increase in interest in the relationship between the gut microbiota and mammalian metabolism has led to the utilization of very elegant and novel molecular techniques based on microbial DNA sequencing [16, 17]. This interest has also brought new insight into the epidemic obesity rates throughout the world and the consequent incidence of type 2 diabetes, comorbidities, and cancer.

Metagenomic analyses of human volunteers showed that almost all bacteria present in the distal gut and feces belong to two main bacterial phyla, *Bacteroidetes* and *Firmicutes* [18]. The predominance of these phyla is also seen in lean mice, with a balance among the bacterial phyla, but in genetically obese *ob/ob* mice, this balance is broken. In obese mice, a great increase in bacteria from the phylum *Firmicutes* and a comparable decrease in the prevalence of *Bacteroidetes* were observed, indicating gut microbiota alteration driven by obesity, predisposing for or associated with this metabolic condition [19]. Moreover, it was demonstrated that microbiota transplantation from *ob/ob* mice to *germ-free* recipients induced a greater increase in body fat content when compared with *germ-free* mice that were the recipients of lean mice gut microbiota, indicating that this difference in the intestinal flora induced the obese phenotype [20]. Additionally, it was shown that the microbiota in the feces of lean and obese humans was different in a similar way as that observed in mice [20]. All this information led to the hypothesis that the gut microbiota from *ob/ob* genetically obese mice is capable of harvesting more energy from the diet [21, 22], probably by the presence and/or increased prevalence of bacteria that produce enzymes that are more efficient in degrading the nutrients available in the diet.

The relationship between the phyla *Bacteroidetes* and *Firmicutes* is the main focus of discussion when obesity is studied, with a considerable amount of data showing an increase in *Firmicutes* prevalence and a reduction in *Bacteroidetes* [19, 20, 23–26]. However, this issue has not yet been completely addressed, as we can find several studies in the literature that maintain the opposite, where the prevalence of *Firmicutes* is decreased in overweight and obese individuals, as well as in obese mice, with an accompanying increase in *Bacteroidetes* prevalence [27–30]. Additionally, the presence of nonsignificant prevalent bacteria phyla in the gut of lean mice, such as *Verrucomicrobia*, has not been well explored until now, with very few observations on increases in pathological conditions (obesity and cancer) and no mechanistic evaluations [27, 31]. The differences observed among gut microbiota human studies still have to be addressed, evaluating the ethnics and feeding behavior of the studied population as well as the standardization of the methods used. In animal studies, the probable cause of the opposite outcomes may be a factor of different mouse strains used. The issue of the “healthy” bacterial group profile is a very important point in gut microbiota research and needs to be further explored. Thus, no proposed treatment attempting to induce the proliferation of certain bacterial phyla has been suggested until now.

## 4. Obesity and High-Fat Diet Increase Circulating Endotoxin Levels and Activation of the Inflammatory Response

**Figure 1:**
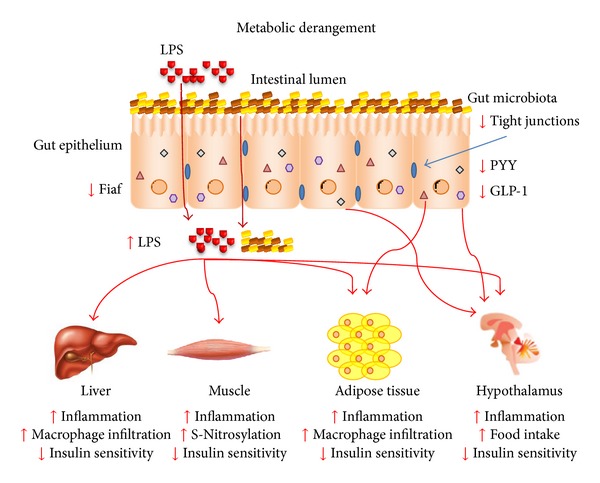
The gut microbiota is modulated by metabolic derangement, such as nutrition overload and obesity, which promote a cluster of metabolic disease-associated processes that culminate in bacterial products and whole bacteria translocation to the circulation through increased intestinal permeability caused by a reduction in tight junction expression. This triggers an immune response, inflammation, and immune cell infiltration of liver and adipose tissue. It induces insulin resistance in various tissues by diverse mechanisms and food intake deregulation in the hypothalamus promoted by the insulin and leptin resistance and also inhibited expression of gut-secreted anorectic hormones, such as GLP-1 and PYY. Additionally, there is a reduction in the intestinal Fiaf expression mediated by bacteria that deregulate the fat storage and lipid metabolism favoring the obese phenotype.

A structural particularity of bacteria splits them into Gram positive and Gram negative, based on the cell wall structure. Each of the two most prevalent phyla belongs to one of these groups; that is, *Firmicutes* are Gram positive and *Bacteroidetes* are Gram negative bacteria. The latter group harbor lipopolysaccharides (LPS) in their cell wall, which is a large molecule formed by a lipid and a polysaccharide that elicits a strong immune response, promoting inflammation to protect the organism from bacterial infection [32]. LPS is a potent activator of pathogen-associated molecular pattern (PAMP) responses, primarily via toll-like receptor 4 (TLR4), which activates an extensive cell signaling pathway that induces the inflammatory response and cytokine expression and secretion [33] (Figure 1).

Several studies have shown that circulating levels of LPS are elevated in obese mice, rats (genetically or induced by diet), and humans. In rodents, this is directly related to increased intestinal permeability [34–36]. This phenomenon occurs due to reduced expression and activity of tight junction proteins, such as zonula occludens-1 (ZO-1) and occludin, that create, together with gut epithelial cells, a barrier that separates the intestinal lumen and its bacterial population and products from peritoneal tissues. This degradation of tight junction function leads to the leakage of bacterial products, such as LPS, and bacterial translocation, which have recently been described as key factors in both human and mice insulin resistance and inflammation [26, 35, 37–39] (Figure 1). Recent evidence also indicates that LPS can be transported along with chylomicrons into the circulation instead of depending on epithelial injury to reach insulin sensitive organs; the inhibition of chylomicron synthesis blocks endotoxin uptake [40].

LPS is a very specific TLR4 ligand with great affinity. Its cell signaling pathway, composed of a diverse number of proteins, culminates with the inflammatory response mediated by LPS contact with cells [41–43]. Beyond the high specificity of TLR4 in identifying LPS from Gram negative bacteria, which can reach insulin sensitive tissues through the circulation from the gut and drive the inflammatory response to protect the host from bacterial infection, TLR4 has a direct role in insulin resistance mediated by obesity. LPS from the gut can react with free-fatty acids (FFA), mostly the saturated type, that are increased in the circulation of obese individuals due to an increase in adipose tissue-mediated lipolysis, *de novo* liver lipogenesis and ectopic lipid accumulation [44]. TLRs have been related to the gut microbiota, whereas single TLR-deficient mice, such as TLR2^−/−^ [26] and TLR5^−/−^ [45] mice, show a different microbiotic profile when compared to their control littermates. This immune modulation of the intestinal flora can induce symptoms of metabolic syndrome, such as increased body weight, blood glucose, and insulin resistance. The TLR4^−/−^ mice do not present an altered prevalence of gut microbiota bacteria as seen in the TLR-deficient mice cited above [46]. In obesity and conditions related to intestinal permeability, TLR4 is known to be involved in the inflammatory response that culminates in insulin resistance and metabolic derangement, as those responses are attenuated by the inhibition of this protein activity [27, 34, 47–49], such as in TLR4 loss-of-function C3H/HeJ mice [50], in CD14^−/−^ mice [51], and in TLR4^−/−^ mice [52] (Figure 1). Recent investigations have shown that fatty acids do not activate TLR4 directly, questioning the real influence of this receptor in lipid-induced insulin resistance caused by obesity [53, 54]. Nevertheless, a hepatic protein has been identified, called fetuin-A (FetA), which is a major carrier of FFAs in the circulation [55], that acts as an endogenous ligand of TLR4, thus activating its signaling pathway, promoting insulin resistance, which was blocked in the absence of FetA, and attenuating insulin resistance induced by FFA [56]. There is evidence that shows increased FetA expression in HepG2 cells, a liver cell lineage, after treatment with thapsigargin, an ER stress inducer, in a time- and dose-dependent manner, which is blocked by pretreating the cells with 4-phenylbutyrate, a compound that inhibits ER stress [57]. These results were similar in diet-induced obese mice, which exhibited ER stress, attenuated by 4-phenylbutyrate treatment and accompanied by a reduction in FetA expression and improved insulin resistance [57]. Additionally, it was demonstrated that in obese individuals and rodents, circulating levels of FetA are increased and correlate with body weight [58, 59] and that body weight loss brings the FetA circulating levels back to normal in children [59]. As well, FetA^−/−^ mice are protected from obesity and insulin resistance induced by aging [60, 61]. 

Other PAMPs, as well as damage-associated molecular patterns (DAMPs), such as the inflammasome seem to be related to intestinal epithelial integrity. The activation of DAMPs and PAMPs in the gut is necessary for the maintenance of barrier function, while nucleotide-binding oligomerization domain protein-like receptor 3 (NLRP3) and NLRP6-deficient mice show increased intestinal permeability and increased risk of colitis [28, 62], allowing the occurrence of dysbiosis and insulin resistance and a greater possibility of nonalcoholic steatohepatitis (NASH). The inflammasome is a group of protein complexes that recognizes a wide range of bacterial, damage and stress signals; it results in caspase-1 activation and subsequent proinflammatory cytokine secretion and cell death [63]. Mice deficient in inflammasome proteins show altered gut microbiota when compared to wild-type littermates, with an increased prevalence of bacteria from the *Prevotellaceae* family, which is part of the *Bacteroidetes* phylum, and higher translocation of bacterial products from the gut to the circulation. In particular, TLR4 and TLR9 agonists induced inflammation and insulin resistance; this feature is transmissible to newborn and adult mice that come in direct contact with inflammasome-deficient animals [28, 62]. In addition to gut microbiota modulation, inflammasome proteins are activated in macrophages by LPS, which enter into the circulation due to increased intestinal permeability under obese conditions. The macrophage inflammatory activity is regulated by the TLR-induced activation of nuclear factor *κ*B (NF-*κ*B) [64, 65].

In addition to the bacterial immune response mediated by TLR4, viral infections also activate pattern recognition receptors (PRRs) and trigger a specific cellular signaling pathway that terminates with the activation of c-Jun N-terminal kinase (JNK), inhibitor of nuclear factor-*κ*B kinase subunit *β* (IKK*β*), NF-*κ*B, and transcription of proinflammatory cytokines, which are mediators of insulin resistance. The double-stranded RNA-activated protein kinase (PKR) is one of the molecules responsible for protection from viral infections, as it identifies double-stranded RNA (dsRNA) viruses and activates the innate immune response against these pathogens [66, 67]. PKR is also related to obesity and insulin resistance, as its phosphorylation is increased in high-fat diet fed mice, leading to activation of JNK [68] and IKK*β* [69], and culminating in serine phosphorylation of IRS-1 and insulin resistance [70]; all these inflammatory features are absent or attenuated in PKR^−/−^ mice [71, 72]. It has been described that PKR is also activated by bacterial products, such as LPS [73, 74], in a dsRNA-independent way, possibly by the action of a cellular protein designated PKR-activating protein (PACT), which possesses dsRNA binding domains and interacts with other molecules that bind to PKR [75] to trigger the same signaling pathway as viral infection [76]. This could integrate the PKR signaling pathway with gut microbiota metabolic effects on insulin resistance, but has not yet been investigated (Figure 1). 

The activation of inflammatory pathways by gut-derived LPS, mainly via TLR4, leads to increased expression of inducible nitric oxide synthase (iNOS) [77, 78]. In obesity, an increase in iNOS expression is also observed in insulin sensitive tissues, which promotes a phenomenon known as S-nitrosation/S-nitrosylation, where nitric oxide (NO) reacts with cysteine residues to form S-nitrosothiol adducts, thereby modulating protein function [79, 80]. Thus, LPS induce S-nitrosation/S-nitrosylation of the insulin signaling pathway (IR, IRS-1, and Akt), inducing insulin resistance in the liver, muscle, and adipose tissue in a more particular way than serine phosphorylation of IRS-1 [81–83]. The targeted disruption of iNOS and its pharmacological inhibition attenuates the S-nitrosation/S-nitrosylation of insulin signaling proteins and inflammation and consequently improves insulin sensitivity [84–87] (Figure 1). Besides gut-derived LPS, other bacteria metabolites could induce S-nitrosation/S-nitrosylation of diverse proteins, modulating their activity and promoting biological effects in several tissues. Therefore, this field needs more attention and further studies.

## 5. Metabolic Role of Short-Chain Fatty Acids Derived from Gut Microbiota

The gut microbiota has an impact on mammalian physiology (mostly metabolic and immune functions) through several mechanisms. Some of them are discussed previously, but the gut bacteria can also interact with the host system through their metabolites, mainly short-chain fatty acids (SCFA), which are their principal end-products and are represented for the most part by acetate, propionate, and butyrate, which have physiological effects in different tissues [88]. Gut microbiota fermentation, due to anaerobic bacteria, degrades polysaccharides that are not cleaved by mammalian enzymes, in other words, nondigestible carbohydrates, to produce short-chain fatty acids and other subproducts in the cecum and colon [89]. Moreover, SCFA diversity demonstrates metabolic cooperation among the bacterial community, as no bacterial genus can hydrolyze all kinds of nutrients nor produce all metabolites found in the gut lumen [90], indicating that most of the physiological interactions of the gut microbiota with the host are not dependent on just one particular bacterial type, but the entire community.

As metabolites, SCFA have diverse effects on various cells. They are taken up by the host through passive diffusion and via mono-carboxylic acid transporters, such as monocarboxylate transporter 1 (MCT1) [91]. Primarily, they work as an energy source for colonic epithelial cells, which derive 60%–70% of their fuel influx from SCFA. Butyrate has a particular importance as an energy source for those cells, as almost 65% is cleared by the mucosa and more than 70% of oxygen consumption in isolated colonocytes is due to butyrate oxidation [89]. Butyrate may also play a critical role in cell growth and differentiation [92, 93]. Acetate is likely to be used as a cholesterol or fatty acid precursor, and propionate is a gluconeogenic substrate [94, 95]. The relative levels of the specific enzymes for SCFA degradation (acetyl-CoA, propionyl-CoA, and butyryl-CoA) in different tissues are the determinants of SCFA metabolization [96]. Other molecules with metabolic regulatory functions can be released by gut bacteria, such as conjugated linoleic acids (CLA) [97, 98], which are lipid metabolites, or bile acids [99] and gases, such as methane and H_2_S [100, 101], but they have minor roles in mammal physiology when compared to SCFA.

SCFA also act as anti-inflammatory molecules, as acetate, propionate, and butyrate are capable of inhibiting NF-*κ*B activation in the host immune cells by binding to G-protein-coupled receptor, 43 and 41 (GPR43 and GPR41), thereby blocking inflammatory responses and suppressing TNF-*α* and IL-6 release; butyrate also reduces IL-12 and increases IL-10 expression [102–105]. GPR43 seems to have great importance in mediating acetate-induced anti-inflammatory stimuli since the deletion of this gene promotes an increased inflammatory response in dextran sodium sulfate- (DSS-) induced colitis, which is attenuated by the administration of acetate in mice that express GPR43 [106]. Moreover, GPR43^−/−^ immune cells are hyperactive and promote an increased response to chemoattractants, such as C5a and proinflammatory cytokines [106].

Acetate and butyrate are also important in epithelial barrier function maintenance as they stimulate the production and secretion of mucus, by goblet cells, which protect the epithelium through increased expression of mucin [107]. Butyrate increases MUC-2 expression by 23-fold in a goblet cell line *in vitro*, demonstrating the importance of this function in modulating intestinal permeability [108]. Butyrate also affects tight junction protein expression, that is, zonulin and occludin, and even low concentrations of this SCFA seem to reduce intestinal permeability [109, 110]. Even with this potent mucus releasing effect by butyrate, it seems that acetate has more pronounced effects in epithelial protection, as activation of GPR43 by acetate protects mice from a lethal infection with an *E. coli* strain [111]. Moreover, the inhibition of GPR strongly attenuates the effects of acetate in terms of epithelial survival and integrity [112].

The products of gut microbiota fermentation, such as acetate and butyrate, are able to increase fatty acid oxidation and energy expenditure. There is evidence that acetate intake by humans promotes a reduction in body weight, circulating cholesterol, and triglyceride levels [113]. The administration of acetate or an increase in gut production mediated by the gut microbiota modulates activated 5′-AMP-activated protein kinase (AMPK), which inhibits acetyl-CoA carboxylase (ACC), thereby promoting fatty acid oxidation and energy expenditure [114] and leading to increased insulin sensitivity and reduced glucose intolerance in diabetic rats and in high-fat diet fed mice [27, 115]. Butyrate administration also increases AMPK activation in muscle, culminating in increased energy expenditure, as observed by an increase in brown adipose tissue mass and UCP1 expression [116].

It has been shown in cell culture experiments that short-chain fatty acids, in particular propionate and butyrate, can modulate the expression of Fiaf in intestinal cells, which was proposed as a gut microbiota mediator of fat storage [14]. Intestinal cell culture lines were exposed to short-chain fatty acids, which promoted an increase in Fiaf expression via PPAR*γ* in colon cells. This was not reproduced in adipocyte cell lines, leading to the inhibition of LPL, adipogenesis and a proposed increase in fat storage mechanism control and metabolic improvement [117–119].

The activity of gut hormones in the control of appetite has been shown [120], and it is likely that the gut microbiota should interfere with the expression and activity of these hormones. Gut bacteria seem to modulate the secretion of glucagon-like peptide-1 (GLP-1), which is produced by L-cells in the colon, and peptide YY (PYY) [121], produced by ileum and colon cells, as well as leptin production by adipose tissue cells via GPR41 [122]. All of these have anorectic effects in the hypothalamus and thus promote satiety. This modulation seems to be, at least in part, mediated by the SCFA produced by fermentative bacteria. Evidence of SCFA and satiety show that an acetate infusion induces an increase in the circulating levels of GLP-1 and PYY in hyperinsulinemic overweight women [123]. Propionate reduces food intake in animal feeding studies [124–126], whereas supplementation of a dairy beverage fermented by propionic acid bacteria producer also increases satiety in humans [127]. In a similar way to propionate, butyrate induces satiety, upregulating anorectic neuropeptide expression, such as PYY and proglucagon in rat epithelial cells [126, 128]. It is possible that these SCFA mechanisms of appetite regulation are mediated by GPR41 and dependent on the intestinal transit rate, as a deficiency in this receptor is associated with reduced PYY expression and faster intestinal transit with reduced energy harvesting from the diet [129]. Most of the observations on SCFA-regulated appetite are descriptive, and the mechanisms controlled by these gut microbiota-derived molecules are not yet known.

## 6. Gut Microbiota Modulation

In the last decade, a great body of evidence and knowledge about the gut microbiota and its interaction with the host, immunity, and metabolism has provided new insight regarding the influence of this forgotten “organ” on the most prevalent metabolic disease, obesity. By several mechanisms, gut bacteria influence the chronic low grade inflammation that culminates in insulin resistance and the increase in fat deposition and body weight gain, characteristic of obese individuals. With the acknowledgement of these obesity and inflammation induction mechanisms, several strategies to block or attenuate them are being developed and tested, in order to benefit obese and type 2 diabetic patients.

### 6.1. Antibiotic Therapy

**Figure 2:**
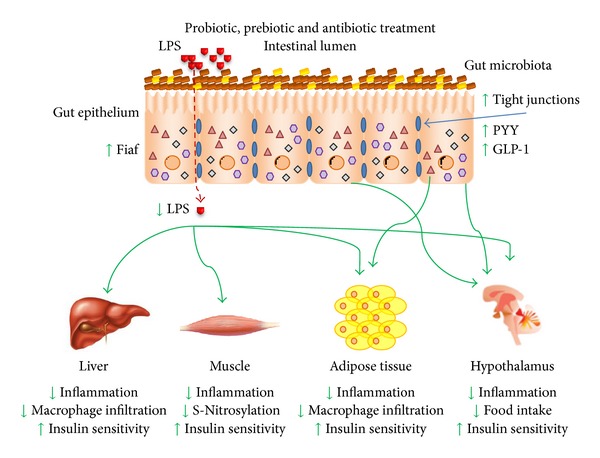
The advent of products (antibiotics, probiotics, and prebiotics) capable of modulating the gut microbiota profile, their products and metabolites (i.e., LPS, short-chain fatty acids), promotes a shift on the bacterial community prevalence, which favored the increase in tight junctions expression and function, reducing intestinal permeability and bacterial products circulation levels. Thus, the LPS circulating levels and inflammatory status in insulin-sensitive tissues are reduced, as well as muscle S-nitrosylation and liver and adipose tissue macrophage infiltration, promoting increased insulin sensitivity and the whole body metabolism. GLP-1 and PYY circulating levels are increased after treatment with gut microbiota modulators which together with the improvement in insulin sensitivity in the hypothalamus promoted reduction in food intake by satiety mechanisms and in conjunction with the increased Fiaf expression, contributed to reduce body weight.

It has been shown that the use of broad spectrum antibiotic therapy greatly modifies the gut microbiota profile in mice, improving the metabolic derangement induced by genetic obesity and/or high-fat diet feeding, but the prevalence of surviving bacteria and the benefits for the host have not been determined, as the concept of a “healthy” gut microbiota is still under investigation. The main mechanism suggested by antibiotic administration is a reduction in circulating LPS levels, which attenuates inflammation and improves the insulin resistance induced by obesity in the liver, muscle, and adipose tissue [27, 35, 130, 131]. Additionally, intestinal permeability may be reduced after the evaluation of tight junction protein expression in epithelial cells of antibiotic-treated animals, showing increased expression and function [35]. This improvement in insulin resistance was also observed in high-fat diet fed mice even in absence of a difference in body weight, which was achieved by submitting a group of animals to pair-feeding in comparison to the antibiotic-treated animals, which presented lower circulating LPS levels and TLR4 activation. This probably led to reduced intestinal permeability, leading to a reduction in JNK and IKK*β* activation and reduced serine phosphorylation of IRS-1 in the liver, muscle, and adipose tissue, altogether seen as increased activation of the insulin signaling pathway, and to inhibition of macrophage infiltration into adipose tissue. Moreover, antibiotic treatment increases the portal acetate levels, which activates AMPK, fatty acid oxidation, and, possibly, energy expenditure [27]. However, even with this striking metabolic improvement in antibiotic therapy experiments, it seems that translating this strategy to humans is not the best option, as there are complex issues such as antibiotic resistance in chronic administration panels and evidence that indicates a relationship between chronic low-dose antibiotic therapy and body weight gain [132, 133] (Figure 2).

### 6.2. Probiotics

Probiotics are defined as live microorganisms that confer unspecified health benefits to the host [134]. Evidence from metagenomic profiles indicates that the obese phenotype shows an increased prevalence of *Firmicutes* [20, 26] in the gut microbiota profile, inferring a negative correlation with metabolism and insulin sensitivity. However, apart from these inconclusive observations on the “healthy” gut microbiota, the most commonly used probiotics are *Lactobacillus*, which belong to *Firmicutes* and *Bifidobacterium* [135]. *Lactobacillus* strain administration leads to several metabolic benefits in both rodents and humans, that is, a reduction in adipocyte cell size and body fat in high-fat diet fed mice [136], a reduction in fat mass and BMI, the promotion of insulin sensitivity [137, 138], and restriction of excessive body weight gain in the first years of life of young children [139]. Although the mechanism by which *Lactobacillus* control excessive adiposity has not been described, changes in fat storage genes expression, such as Fiaf, have been proposed to mediate this probiotic effect [140]. Data from *Bifidobacterium* administration show that the production of acetate mediates gut epithelial integrity and barrier function, protecting animals from a lethal bacterial infection [111], but no mechanism as to how acetate induces this effect has been proposed. It is tempting to suggest that acetate binding to GPR43 and its activation mediate the beneficial effects of treatment with *Bifidobacterium*.

A probiotic compound that has been well studied and appears in a great number of articles, named VSL#3, composed of a blend of probiotic bacteria, modulates the gut microbiota profile [141] and shows interesting effects, such as the promotion of epithelial integrity by modulation of tight junctions [142], a reduction in inflammatory status in a chronic colitis animal model [141], protection against DSS-induced colitis, and near reversal of atherosclerotic lesions in ApoE^−/−^ mice, which develop atherosclerosis spontaneously [143]. In a clinical trial, VSL#3 promoted reduced intestinal permeability and improved immune activity [144]. Bacterial translocation to mesenteric adipose tissue, which seems to precede type 2 diabetes onset, is prevented by probiotic treatment. Apparently, this mechanism is mediated by acetate production and increased gut epithelial integrity [39]. It has also been shown that probiotic treatment-induced gut microbiota modulation can suppress high-fat-diet induced NF-*κ*B activation and inflammation-induced insulin resistance [145]. Probiotics can also mimic commensal bacteria and modulate protective epithelial mucus production, reduce bacterial adhesion, increase tight junction expression, enhance epithelial and immune cell survival, induce defensins, and stimulate IgA production as well as stimulate TLRs to promote gut homeostasis [146–148] (Figure 2).

### 6.3. Prebiotics

Prebiotics are defined as a nonviable food component that confers a health benefit on the host associated with gut microbiota modulation [149]. The most common prebiotics used in gut microbiota modulation studies are the inulins, fructooligosaccharides, various types of galactooligosaccharides, and resistant starches. Prebiotics act by modulation of the gut microbiota profile and serve as a substrate for the production of metabolically active metabolites, in particular, SCFA, that are, acetate, propionate, and butyrate [150, 151]. Several studies have related prebiotic treatment to a reduction in ectopic lipid accumulation such as steatosis, reduced fat storage in white adipose tissue, in systemic inflammation, and insulin resistance in high-fat diet fed and genetically obese models [152–154], and also reduced endotoxemia [155]. In clinical experiments, beneficial effects of prebiotic administration were observed, such as a reduction in BMI, waist circumference, fat mass, and insulin resistance [156–158]. The food intake regulation is another important feature of gut microbiota modulation by prebiotics, which induce gut hormone production, such as GLP-1 and PYY, that signal via anorectic pathways in the hypothalamus, and a reduction in ghrelin expression, a gastric orexigenic peptide, thereby reducing food intake [159, 160]. Even fibers that do not change the gut microbiota profile, in a similar way to inulin-type fructans, induce food intake reduction by increasing circulating levels of GLP-1 and PYY [161–165], evidencing the important role of SCFA derived from prebiotic fermentation. Data from a 12-week prebiotic treatment in obese subjects showed a modulated gut microbiota profile, increased PYY, and decreased ghrelin circulating levels, while a single dose of prebiotics (inulin) also decreased plasma ghrelin and increased GLP-1 [166, 167]. Prebiotics also induce the production and secretion of GLP-2 by L cells; this hormone has relevant activity on gut barrier function and reduces gut permeability in obese animals [168] (Figure 2).

### 6.4. Bariatric Surgery

Recent data on obese subjects who have undergone gastric bypass surgery indicate that six months after the procedure, the gut microbiota profile was changed and bacteria diversity was reduced in comparison with the subjects that were obese and did not undergo the surgical procedure [169]. In addition to the changes promoted by gastric bypass, physiologically and anatomically, a number of factors could contribute to this gut profile alteration throughout this six months, primarily food intake behavior and weight loss, placing the direct influence of bariatric surgery in doubt. An early evaluation of the gut microbiota after the procedure is not viable due to the antibiotic treatment indicated for surgical recovery. Nevertheless, a recent study brought new light to this issue, where mice that underwent gastric bypass surgery presented a distinct gut microbiota profile when compared with the sham-operated mice one week after of the procedure, when no body weight difference was detected. Furthermore, the authors placed a group of mice under diet restriction to mimic the weight loss achieved by the gastric bypass group for 10–12 weeks and found that the microbiota profile was different as well, indicating that, indeed, the bariatric surgery modifies the bacterial composition of the gut [170]. In addition, they showed that gut microbiota transplantation from a sham-operated animal to *germ-free* mice increased adiposity as well as circulating leptin levels concomitantly with reduced food intake, as was previously demonstrated [14]. However, in the recipients of gut microbiota from mice that underwent gastric bypass, none of those parameters were altered when compared to *germ-free* mice. There was also a reduction in body weight, suggesting that the gastric bypass-associated gut microbiota may either reduce energy harvesting from the diet or produce signals to regulate energy expenditure and/or lipid metabolism in a Fiaf-independent manner since colonization of *germ-free* mice with either sham or gastric bypass gut microbiota inhibits Fiaf expression in intestinal cells, but this is still unknown [170]. Based on the studies cited previously, gastric bypass seems to promote gut microbiota modulation that induces beneficial effects in obese humans and rodents.

## 7. Conclusions

In summary, the literature presents incontestable data that the gut microbiota and its modulation by nutrients and excessive energy intake heavily influence glucose metabolism, lipid storage, inflammation, and insulin activity in a negative way, as observed in obesity and diabetes. Furthermore, there are intense investigations going on in this field, highlighting several strategies to modulate the gut microbiota profile to a “healthier” status in an attempt to increase insulin sensitivity by blockage of the insulin resistance inductors mediated by the interaction between bacteria and the intestinal environment of the host.

Some issues have been raised from these studies and have not been fully explored until now, such as PKR activity modulated by gut microbiota. Besides bacterial products, viruses are also found in metagenomic profiles, and viral signaling could contribute to the increased inflammation promoted by intestinal products in obesity. In a similar way, the nitrosative contribution from gut microbiota has not yet been raised as having an impact on the metabolic derangement mediated by obesity and microbial products. Also, it is unknown if attempts to ameliorate the metabolic status by gut microbiota modulation will regulate both phenomena. SCFA seem to have an important role in food intake regulation, but the mechanisms of inducing GLP-1 and PYY production in intestinal cells are still poorly described and need further investigation. Saturated fatty acids are known to promote inflammation and insulin resistance and are prevalent in the high-fat diet administered to experimental rodents in these studies and in the western diet that has led to epidemic rates of obesity. However, the utilization of unsaturated fatty acid supplementation, which is known as a lipid with anti-inflammatory properties, has not yet been related to gut microbiota modulation. This is an interesting nutritional field, as probiotic and prebiotic strategies can have irrefutable benefits to the host's metabolism.

Ongoing efforts will try to determine the best set of symbiotic bacteria for mammals to harbor in the intestine in a metabolic evaluation, avoid excessive energy uptake from the diet, preserve gut barrier function, and reduce bacteria and the translocation of their inflammatory products; it is hoped that this will culminate in reduced inflammation. This issue is under debate, as articles do not show coherence regarding bacterial prevalence modulation in the gut induced by obesity and its treatment, which will need further studies and standardization. It seems that studies from different facilities bring distinct results. This issue has to be addressed in order to define what bacterial colony is more interesting to have grown in the mammalians gut.
